# Experience with video‐assisted thoracoscopic placement of pectus excavatum splints in kittens: six cases

**DOI:** 10.1111/jsap.70142

**Published:** 2026-05-10

**Authors:** G. Jones, Z. Peng, S. Gasson, R. Williams, K. T. Mankin, V. Dickerson

**Affiliations:** ^1^ Department of Veterinary Small Animal Clinical Sciences College of Veterinary Medicine and Biomedical Sciences, Texas A&M University College Station Texas USA; ^2^ Department of Veterinary Small Animal Clinical Sciences College of Veterinary Medicine, University of Florida Gainesville Florida USA

## Abstract

**Objectives:**

This case series aims to describe the use of video‐assisted thoracoscopic surgery for the treatment of pectus excavatum.

**Materials and Methods:**

Six male, intact kittens, with a median age of 3.3 months (range, 2.5 to 4 months) and median weight of 1.75 kg (range, 0.44 to 2 kg), were presented to one of two referral institutions for evaluation of a concave sternal deformity. On presentation the kittens were found to have radiographically moderate (*n* = 3) to severe (*n* = 3) pectus excavatum of the caudal sternum with mild to moderate respiratory signs reported at home. Surgical correction included the use of video‐assisted thoracoscopic surgery to facilitate external sternal splint placement. Use of video‐assisted thoracoscopic surgery allowed for direct visualisation of needle passage to place the circumsternal sutures, potentially reducing the risk of complications given the proximity of intrathoracic vasculature and organs.

**Results:**

All patients recovered from anaesthesia uneventfully, and post‐operative thoracic radiographs revealed reduction in sternal concavity and increase in thoracic height at the level of the original deformities. One cat suffered respiratory arrest 36 hours post‐operatively for unknown reasons.

**Clinical Significance:**

Video‐assisted thoracoscopic surgery‐assisted external splint placement for pectus excavatum may help reduce intraoperative risks associated with pectus excavatum repair, though additional research is warranted.

## INTRODUCTION

Pectus excavatum (PE) is a congenital deformity described in both dogs and cats characterised by a dorsal deviation of the sternebrae and associated costal cartilages (Charlesworth et al., 2016; Crigel & Moissonnier, 2005). The deviation results in dorsoventral narrowing of the thoracic cavity that can result in cardiopulmonary compression (Charlesworth et al., 2016; Crigel & Moissonnier, 2005). A genetic component has been suggested, as a breed predisposition has been reported in Bengal and Burmese cats (Charlesworth & Sturgess, 2012; Sturgess et al., 1997; Venzo & Libermann, 2025).

Diagnosis of PE is primarily based on physical examination, the presence of clinical signs, and radiographic assessment. The most common clinical signs include tachypnoea, exercise intolerance, dyspnoea, cardiac murmur, cyanosis and arrhythmia (Charlesworth et al., 2016; Yoon et al., 2008). The anatomical severity of PE can be determined radiographically by calculating the frontosagittal (FSI) and vertebral indices (VI) (Charlesworth, 2017; Charlesworth et al., 2016; Komsta et al., 2022). A normal FSI ranges from 0.7 to 1.3 while a normal VI ranges from 12.6 to 18.8. Calculating the VI and FSI can help determine anatomical severity but does not necessarily correlate to the degree of clinical signs (Charlesworth et al., 2016; Hassan et al., 2018).

Treatment of PE in animals diagnosed at a young age typically involves the application of an external sternal splint secured to the chest by the placement of circumsternal sutures (Charlesworth, 2017; Crigel & Moissonnier, 2005). The sutures place ventral traction on the dorsally deviated sternebrae allowing for correction of the deformity as the animal grows (Charlesworth et al., 2016). This technique works in young animals because the sternum and costal cartilage are pliable, which allows for reshaping of the thorax when ventral traction is placed on the sternum by the external splint (Crigel & Moissonnier, 2005; Fossum et al., 1989; Yaygingul et al., 2016).

Complications associated with PE splint placement are uncommon, but may include pulmonary oedema due to re‐expansion, iatrogenic damage to the heart, lungs and thoracic vasculature during the blind passage of the needle around the sternum, and iatrogenic pneumothorax (Charlesworth, 2017; Soderstrom et al., 1995). Video‐assisted thoracoscopic surgery (VATS) has been evaluated for the treatment of PE in a 2022 canine case report and a 2025 feline case report with the goal of minimising iatrogenic injury (Bobis Villagrà & Charlesworth, 2022; Venzo & Libermann, 2025). This technique may reduce the risk of intraoperative complications by allowing direct visualisation of the needle as it passes near intrathoracic structures. The aim of this case series was to describe the use and report the efficacy of VATS to repair PE in kittens.

## MATERIALS AND METHODS

Six kittens were referred to either Hospital A or Hospital B for evaluation of presumed PE (Table 1). All kittens were intact males. Breeds represented included domestic shorthair (*n* = 2), domestic longhair (*n* = 2), Persian (*n* = 1) and Maine Coon (*n* = 1). Median age was 3.3 months (range, 2.5 to 4 months). Median weight was 1.75 kg (range, 0.44 to 2 kg). On physical examination, Cats 1 to 3 had moderate, palpable concave sternal defects and were clinically normal at rest; however, mild respiratory signs were reported in association with activity or certain body positions. Cats 4 and 5 both had severe, palpable caudal sternal deformities with similar radiographic findings. However, Cat 4 exhibited dyspnoea associated with stress, while Cat 5 showed dyspnoea and tachypnoea even at rest. Cat 6 presented through the emergency service for dyspnoea, tachypnoea and lethargy, and had a severe palpable caudal sternal deformity. The remainder of the physical examination and bloodwork were unremarkable for all six kittens. A detailed summary of clinical signs is provided in Table 1.

**Table 1:** Signalment, clinical signs, frontosagittal index (FSI) and vertebral indices (VI) of four kittens that underwent video‐assisted thoracoscopic splint placement for pectus excavatum

Cat no.	Age (months)	Weight (kg)	Clinical signs	FSI			VI		
				Pre‐splint	Post‐splint	At splint removal	Pre‐splint	Post‐splint	At splint removal
1	3	1.9	Positional dyspnoea	2.99	1.93	1.87	6.12	8.35	10.75
2	4	1.7	Tachypnoea with normal effort	3.04	1.58	1.36	6.67	9.54	10.80
3	4	2.0	Dyspnoea and tachypnoea with increased activity	2.37	1.27	1.24	6.47	9.20	10.20
4	3	1.6	Dyspnoea when stressed	3.3	1.36	1.31	5.35	12.30	11.94
5	3.5	1.8	Dyspnoea and tachypnoea at rest	7.43	2.46	1.80	3.58	8.22	8.80
6	2.5	0.44	Dyspnoea, tachypnoea, lethargy	3.62	1.96	N/A	5.43	8.79	N/A

Normal FSI is between 0.7 and 1.2, and normal VI is between 12.6 and 18.8

Radiographic evaluation revealed dorsal deviation of the caudal sternebrae, resulting in cranial and leftward deviation of the cardiac silhouette in all kittens. The anatomical severity of PE was assessed by using the frontosagittal (FSI) and vertebral indices (VI). Briefly, the FSI is the ratio between the thoracic width at the level of the 10th thoracic vertebra and the distance from the ventral aspect of the 10th vertebra or vertebra overlying the deformity to the nearest point on the sternum. The VI is the ratio between the distance of the thorax at the centre of the dorsal surface of the vertebral body overlying the deformity, to the nearest point on the sternum and the dorsoventral diameter of the vertebra at the same level (Charlesworth, 2017). Based on FSI and VI values, Cats 1 to 3 were classified as having moderate sternal deformities, with frontosagittal index (FSI) values ranging from 2.37 to 3.04 and vertebral index (VI) values from 6.12 to 6.67. In contrast, Cats 4 to 6 exhibited severe deformities, with FSI values ranging from 3.30 to 7.43 and VI values from 3.58 to 5.43 (Table 1). Owners of the kittens were counselled on the pros and cons of conservative management or pursuing splint placement, with the primary benefit being the potential to manage the deformity with a splint alone at a young age, *versus* the potential for a more aggressive surgery if the deformity persists or progresses as the kittens aged. All owners were made aware that the kittens' mild signs could improve with no treatment as the kittens aged. All owners were also informed of both VATS‐assisted splint placement and blind splint placement options. All elected to move forward with VATS‐assisted splint placement.

All kittens were monitored by a board‐certified anaesthesiologist while under anaesthesia. Cat 1 was premedicated with midazolam (0.2 mg/kg, iv) and alfaxalone (2 mg/kg, iv); Cat 2 with methadone (0.18 mg/kg, im) and alfaxalone (2 mg/kg, im); Cat 3 with methadone (0.5 mg/kg, im) and dexmedetomidine (1 mcg/kg, im); Cat 4 with methadone (0.18 mg/kg, im), dexmedetomidine (15 mcg/kg, im), alfaxalone (2 mg/kg, im) and telazol (2 mg/kg, im); Cat 5 with methadone (0.5 mg/kg, im), alfaxalone (2 mg/kg, im), and dexmedetomidine (4 mcg/kg, im); and Cat 6 with methadone (0.2 mg/kg, im), alfaxalone (4 mg/kg, im), and midazolam (0.2 mg/kg, im). General anaesthesia was induced with propofol (up to 6 mg/kg, iv) and ketamine (1 mg/kg, iv) in Cats 1 and 2, with propofol (3.5 mg/kg, iv, 2 mg/kg, iv, and 4 mg/kg, iv, respectively) in Cats 3 to 5, and alfaxalone (2.7 mg/kg, iv) in Cat 6. All patients were maintained with sevoflurane in 100% oxygen *via* mechanical ventilation. Fluid therapy (Lactated Ringers Solution 3 mL/kg/hour) was administered during the intraoperative period. Cefazolin (22 mg/kg iv) was administered before the operation and every 90 minutes for the duration of anaesthesia. Glycopyrrolate (0.01 mg/kg iv) was administered intraoperatively in Cats 1 and 2 for bradycardia and hypotension. Ketamine (20 mcg/kg/minute, iv) was administered intraoperatively and atropine (0.01 mg/kg, iv) was administered post‐operatively for bradycardia and hypotension in Cat 6.

**FIG 1:**
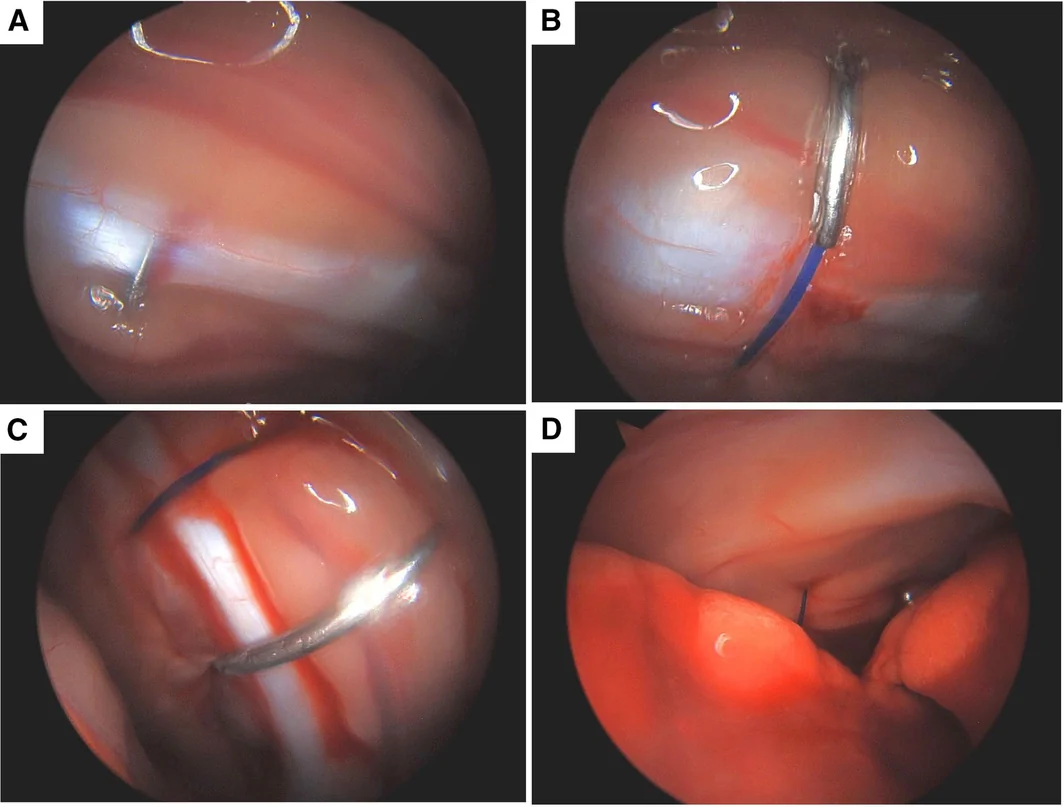
Thoracoscopic images acquired during circumsternal suture placement for pectus excavatum in a kitten. (A) The needle passing through, rather than around the sternebra. (B) Correction of needle direction and successful circumsternal suture placement. (C) Residual mild haemorrhage from puncture of an internal thoracic artery during placement of a prior suture. (D) The proximity of the lungs to the sternum.

The cats were placed in dorsal recumbency with the right side as close to the edge of the table as possible, to permit range of motion with the arthroscope. The ventral thorax and cranial abdomen were clipped and prepped with alternating chlorhexidine gluconate and isopropyl alcohol and draped in routinely. Given a leftward deviation of the heart in all cats, a right‐sided approach was elected. A stab incision approximately 3 mm in length was made in the dorsal third of the third to fifth intercostal space on the right thoracic wall. Blunt dissection of the soft tissues was achieved with Metzenbaum scissors and a mosquito haemostat, and a pneumothorax was induced. In Cats 1 to 3, a 30°, 4 mm‐diameter, 140 mm‐length Arthrex arthroscope was inserted directly into the thoracic cavity through the stab incision made in the third to fifth intercostal space, without a cannula (Arthrex Inc., Naples, FL, USA). In Cats 4 to 6, a 30°, 2.7 mm‐diameter, 72 mm‐length Arthrex arthroscope was utilised. The thoracic cavity was visualised (Arthrex Inc., Naples, FL, USA). The xiphoid was grasped externally with a towel clamp or directly with a circumxiphoid suture (2‐0 polypropylene) to elevate the sternum. The surgeon held the camera standing on the right side of the patient, while an assistant stood either on the patient's left side or at the patient's pelvic limbs to pass the sutures under direct visualisation. Beginning at the xiphoid and moving cranially, a total of 5 to 8 circumsternal sutures were placed 10 to 20 mm apart using 0 polypropylene suture on a taper‐point CT‐1 needle for Cats 1 to 5 and 20 lb nylon leader line in Cat 6. The suture tags were left long and secured with a mosquito haemostat. In Cats 1 and 2, the needle was frequently noted to go through the sternebrae rather than around them (Fig 1A, B). It was not always possible to adequately adjust the needle such that it passed circumsternally, thus some sutures were left through the sternebra in three cases. Puncture of an internal thoracic artery occurred during one needle passage in Cats 1 and 2, but haemostasis was achieved when pressure was placed on the suture (Fig 1C). Working space provided by the pneumothorax allowed the heart and lungs to shift dorsally, away from the sternum, preventing inadvertent puncture of these structures (Fig 1D). After placement of all sutures, an 18 gauge over the needle intravenous catheter was temporarily passed into the thoracic cavity at the seventh intercostal space under direct visualisation with the scope to permit evacuation of air after incision closure. The arthroscope was removed from the thoracic cavity. The scope entry site was closed with a single interrupted 4‐0 Monocryl suture in the subcutis followed by a single intradermal suture. All air was evacuated from the thoracic cavity using the previously placed intravenous catheter, which was subsequently removed following evacuation of air.

A sheet of prepared thermoplastic mesh (Orfit Classic Mini Perforated 3.5%, 3.2 mm thickness) was conformed to the desired shape of each kitten's thorax and cut to size in Cats 1 to 5, and a sheet of pre‐formed polyurethane resin impregnated with fibreglass tape (Vetcast, 3M) was used for Cat 6 (Orfit Ind., Norfolk, VA, USA; 3M, St. Paul, MN, USA). Additional holes were added to the splint as needed using a 16‐gauge needle pushed through with a needle driver. The ends of the sternal sutures were passed through the pre‐drilled holes. Gentle traction was applied to deviate the sternum ventrally to the splint and the sutures were secured with split shot in Cat 1 and tied in Cats 2 to 6.

## RESULTS

Immediate post‐operative radiographs revealed improved FSI and VI in all cases (Table 1). In Cat 1, initial radiographs revealed that the reduction of the sternal defect was insufficient at the caudal‐most aspect. Therefore, additional holes were made in the splint and a single suture was blindly placed percutaneously at the caudal‐most aspect of the defect with the splint remaining on the patient while manoeuvring around the end of the splint. This provided additional sternal traction and alignment at the level of the xiphoid. Radiographs were taken after the addition of the suture and revealed a more desired reduction in the sternum. Surgical tape and a stockinette were used to cover the splint. While under anaesthesia, Cats 1, 3, and 4 also underwent a routine scrotal castration. Median surgical time (from incision for port placement to completion of splint application) was 79 minutes (range, 45 to 90 minutes). All six recovered from anaesthesia uneventfully.

Post‐operative pain was managed with gabapentin (*n* = 3), buprenorphine (*n* = 3), robenacoxib (*n* = 1), fentanyl CRI (*n* = 1) and methadone (*n* = 1). Cat 1 was discharged 2 days post‐operatively, while Cats 2 to 5 were discharged 1 day post‐operatively. Cats 1 to 5 kittens had a normal respiratory rate and effort at the time of discharge. Approximately 36 hours post‐operatively, Cat 6 became hypothermic (97.4 °F) and hyporexic. Point‐of‐care ultrasound revealed no pleural effusion nor pneumothorax. While attempting to collect a venous blood sample, the patient went into respiratory arrest; cardiopulmonary resuscitation was attempted but was unsuccessful. A necropsy was not performed.

All five remaining kittens tolerated the splints well. Weekly rechecks were performed for the initial 4 weeks to reassess the splint and adjust as needed. The splint remained in place for a median of 6 weeks (range: 4 to 8 weeks). Further improvement in FSI and VI since the time of splint placement compared to splint removal was noted in 4/5 cats (Table 1). All cats developed mild moist dermatitis underneath the splint which resolved following splint removal. No pre‐operative respiratory signs were noted, and no clinical concerns were reported during the post‐operative weekly splint rechecks. Following splint removal, Cat 1 returned for two additional rechecks at 2 weeks and 4 weeks post‐splint removal with no complications reported. Cats 2 to 5 were not evaluated after splint removal. At 10 months post‐operatively, the owner of Cat 3 reported the cat was doing well at home, with one self‐limiting episode of dyspnoea and tachypnoea. Cat 2 was reported to be doing well at 12 months post‐operatively with no clinical signs noted but died several months later due to an unrelated traumatic event. The remaining cats were lost to long‐term follow‐up.

## DISCUSSION

This case series details the use of VATS in the surgical repair of PE in cats. External splinting was elected for the cats described here as feline sternebrae remain pliable in patients under 4 months old (Yoon et al., 2008). Traditionally, sutures are passed blindly, carrying risks such as damage to intrathoracic organs, vasculature and incomplete sternal encircling (Bobis Villagrà & Charlesworth, 2022; Mestrinho et al., 2012). In animals with severe PE, the sternum comes in closer contact to intrathoracic structures and vasculature, which may increase the risk for iatrogenic damage and insufficiently placed sutures (Charlesworth et al., 2016). In these more severely affected patients, VATS may be particularly beneficial by allowing direct visualisation of intrathoracic structures during suture placement, thereby potentially limiting iatrogenic injury and improving accuracy; however, further research is needed to understand whether it provides an advantage over traditional blind suture placement.

In Cats 1 and 2 of the present report, the internal thoracic artery was punctured. This was visualised and assessed thoracoscopically to confirm haemostasis, which occurred by placing pressure on the suture. It was likely this occurred during blind placement but resolved when the sutures were tightened, as observed thoracoscopically. The pneumothorax created during thoracoscopic access allowed the heart and lungs to displace dorsally, which likely reduced the risk of iatrogenic injury to these structures during needle passage. However, despite direct visualisation, precise control of needle trajectory remained challenging, and visualisation was intermittently obscured by lung ventilation. Thoracoscopic imaging also revealed that the initial needle passage only partially encircled the sternebra for some suture passes. Importantly, puncture of the internal thoracic artery still occurred, indicating that VATS does not eliminate the risk of vascular injury. Modifications in needle selection, such as differences in needle curvature or size, may improve the ability to achieve consistent circumsternal placement, though this warrants further investigation. It is not known whether the strength of a true circumsternal suture surpasses that of one that passes through a sternebra. Additionally, long‐term complications related to damage to the sternebrae in cats following splint placement for PE have not been reported to the authors' knowledge. No sternal fractures were identified on post‐op radiographs in Cats 1 and 2, suggesting that passage through the sternebrae did not result in clinically apparent structural damage in these cases.

While the use of VATS allows for thoracic cavity visualisation, it is important to note that the xiphoid extends into the abdomen. Therefore, a blind suture through the most caudal aspect of the deformity will still be necessary. In Cat 1, post‐operative radiographs revealed that initial sternal reduction was suboptimal; when visualising this thoracoscopically, the caudal‐most suture was just cranial to the diaphragm, leading the surgeon to believe the entire defect was traversed. An additional suture was blindly placed in the caudal‐most aspect of the defect around the xiphoid to improve overall reduction after radiographs were taken. However, given the application of the splint on the patient, desired placement of the suture was challenging, emphasising the need to place this suture prior to splint placement.

For all six cats, adequate suture placement was ultimately achieved; however, the size of the scope posed a challenge when trying to achieve optimal visualisation. A 4.0 mm‐diameter, 140 mm‐length arthroscope was used in three of the cases; however, the patients' small size occasionally hindered the ability to freely manoeuvre the scope within the thoracic cavity. A 2.7 mm‐diameter, 72‐mm‐length scope was used in the remaining three cases. Given the shorter length of this scope, it is particularly important to position the patient as far lateral as possible, such that the camera does not hit the table when manoeuvring the scope. The authors' recommend using a 30° scope. This angle allowed adequate visualisation of the sternebrae with the fifth intercostal placement. Ultimately, blind placement of some sutures may still be necessary due to lack of ability to visualise with the scope. Additionally, this technique may provide an accessible alternative for surgeons who do not have access to a needle‐scope, as described in a recent feline case report, thereby broadening the applicability across different practice settings (Backman & Marvel, 2025).

One cat died 36 hours post‐operatively; a necropsy was not performed to determine the cause. Fatal re‐expansion pulmonary oedema following PE splint placement has been reported; however, this is typically recognised immediately following splint placement while still under anaesthesia, thus seems unlikely to be the source in this case (Soderstrom et al., 1995). Pre‐procedural point‐of‐care ultrasound (POCUS) and cardiac evaluation revealed no significant abnormalities. A POCUS examination was also performed when the patient decompensated post‐operatively with no abnormalities reported. However, a progressive or recurrent pneumothorax cannot be ruled out, particularly given that an indwelling chest tube was not placed. Additionally, this patient presented with the most severe clinical signs at admission, raising the possibility of underlying cardiopulmonary or systemic disease contributing to outcome. Changes to the lungs or heart may have been obscured radiographically due to the PE deformity. Without necropsy examination, it is not possible to determine if this death was directly related to the VATS approach or not.

Although non‐surgical management has been proposed as a viable treatment option for asymptomatic or mild cases in dogs, its effectiveness in cats has not been well established and may vary due to species differences (Rahal et al., 2008). As five of the cases did not present with severe clinical signs, the owners were informed of both conservative management and surgical correction options. The benefits and risks of splint placement were discussed (Rahal et al., 2008). Given that the success of splint placement decreases with age due to decreased pliability of the sternebrae and that both owners perceived the mild signs at home to be unacceptable, it was elected to move forward with splint placement (Yoon et al., 2008). Additional research following untreated cases of moderate PE over time would be beneficial to provide further guidance on the outcome without splint placement, as splint placement may not have been necessary in these cases.

While VATS allows for direct visualisation, potentially reducing the associated intraoperative risks and improving surgical outcome, it requires advanced skills of both the surgery and anaesthesia teams. Though non‐VATS surgical times were not available for comparison, use of VATS guidance is likely associated with an increased surgery/anaesthesia time. Prospective research comparing traditional *versus* VATS‐assisted PE splint placement is needed to better understand potential advantages or disadvantages of this technique.

In conclusion, the application of VATS for PE external splinting is feasible, safe, and may be a beneficial adjunct to PE splint placement in feline patients. In this case series, VATS provided direct visualisation of intrathoracic structures, allowed confirmation of haemostasis following vascular puncture, and may have reduced the risk of iatrogenic injury to the heart and lungs. However, the use of VATS can be a technically challenging procedure even in moderately affected kittens and did not eliminate the risk of vascular injury or ensure consistent circumsternal suture placement. VATS may be most beneficial in patients with more severe deformities, where the proximity of the sternum to intrathoracic structures increases the risk of iatrogenic injury and complicates accurate suture placement. Further studies comparing traditional and VATS‐assisted techniques, particularly in severely affected patients, are warranted to better define its clinical utility.

### Author contributions

G. Jones compiled all data, assisted with radiograph scoring, drafted and revised the manuscript. Z. Peng assisted with radiograph scoring and manuscript revision. S. Gasson, R. Williams, and K. T. Mankin contributed case data and assisted with manuscript revision. V. Dickerson conceptualised the study, contributed case data and revised the manuscript.

### Conflict of interest

The authors declared no potential conflicts of interest with respect to the research, authorship, and/or publication of this article.

### Funding

The authors received no financial support with respect to the research, authorship, and/or publication of this article.

## Data Availability

The data that support the findings of this study are available from the corresponding author upon reasonable request.
